# Notes and News

**Published:** 1894-09-15

**Authors:** 


### Notes and News.

Collected and Communicated.

The Metropolitan Hospital Saturday Fund Collection for this year has now reached the sum of nearly £12,000.

Sir Henry Calcraft (late Secretary to the Board of Trade) has accepted a seat on the Board of Management of the Chelsea Hospital for Women, Fulham Road, London, S.W.

The next annual meeting of the British Medical Association will be held in London, and the next meeting of the International Congress of Hygiene and Demography will be at Madrid.

A sad accident happened at Derby last week in connection with the demolition of the old infirmary. Whilst some workmen were employed on the structure a floor gave way, killing three men and injuring others severely.

In the designs for monuments a striking lack of the sense of humour frequently shows itself. This is very apparent in that wonderful arcade of monuments at the cemetery at Genoa. A long corridor is lined with life-sized representations of the departed, executed for the most part in exquisite marble, and although some of these monuments are of beautiful design, a large number are so faithful to the costume worn by the deceased in life, that the solemnity of the scene is entirely overcome. Even a patch on a boot, and a darn in a shawl, find a faithful portraiture in lasting marble, and one realism after another provokes a smile as one passes down the avenue of monuments. It seems as if the memorial to be erected to a medical hero, Surgeon Parke, at Dublin, promises to be unnecessarily realistic. Surgeon Parke is to be represented dressed in his African garb, leaning on a gun, whilst his left foot rests on a medicine chest. For some reason or other the medicine chest of Surgeon Parke is supposed to have particular interest for the public. It is now figuring at the Antwerp Exhibition, and could it be "embalmed," might be perpetuated for ever in a still more real manner, by taking its place *in propriâ personâ* under the African hero's foot.

The "Caledonia" was, by the kind permission of the P. and O. Company, shown to visitors last week on payment of one shilling, the proceeds of which are for distribution amongst the Seamen's and Dock Hospitals.

The cholera is not yet showing much diminution in Russia. From August 19th to September 1st, 1,383 cases with 640 deaths were recorded in from the Government of Warsaw alone. A few isolated cases are reported from Holland and Belgium. One ship from Antwerp has been put in quarantine at Belfast, a death from cholera having occurred on board.

A French contemporary points out the discrepancy between French and American statistics relating to lady doctors. At the beginning of the present year only sixteen lady students in medicine presented themselves at the Paris School of Medicine. It will be long, one contemporary points out, before France can count her 2,000 lady doctors.

If microbes are not exterminated in Russia, it will not be for want of conscientious effort to discourage them. We now hear that a society for the suppression of hand-shaking has been established at Baku, in Russia. The renunciation of this custom will not be quite as difficult as that of kissing, but should it spread to England, we shall become more awkward in our salutations than ever, whilst microbes we fear, will still reign triumphant in spite of all.

The antitoxin treatment, which is one of the special subjects considered at the International Congress of Hygiene, is likely to be actively taken up in America. Dr. Briggs has returned to New York after studying the method at Berlin, and through his report the New York Board of Health are asking for an appropriation to enable them to manufacture the diphtheria antitoxin in large quantities, and thus permit it to be sold at a sufficiently low price to bring it within the means of the poor.

An excellent scheme is being placed on foot in France under the initiative of Mons. Marcel Baudouin. This is a medical circulating library. The absence of contemporary medical literature from the *entourage* of most country doctors is a noticeable fact. The absence is largely the result of necessity, for medical works are mostly expensive. The creation of a really representative circulating library would be a great benefit towards those who would keep on a level with the progress of their day, whilst those more content with the knowledge acquired in student days, followed by personal experience, would nevertheless be stimulated to more intelligent practice if good works could be perused for trifling outlay. Apparently for some, useful as they undoubtedly are to most, the contemporary literature of the medical papers does not stimulate the self-satisfied and unenterprising of the profession to study. We have seen whole piles of one of the medical journals lying unopened in a corner of the room of a medical man who certainly came across no other form of medical literature, and who was under the impression that he could not afford to buy medical books, which were constantly being superseded. A circulating library might, at any rate, meet such cases.

